# ChlamBase: a curated model organism database for the *Chlamydia* research community

**DOI:** 10.1093/database/baz041

**Published:** 2019-04-15

**Authors:** Tim Putman, Kevin Hybiske, Derek Jow, Cyrus Afrasiabi, Sebastien Lelong, Marco Alvarado Cano, Gregory S Stupp, Andra Waagmeester, Benjamin M Good, Chunlei Wu, Andrew I Su

**Affiliations:** 1 Ontology Development Group, Library, Oregon Health and Science University, Portland, OR, USA; 2 Division of Allergy and Infectious Diseases, Department of Medicine, University of Washington, Seattle, WA, USA; 3 Department of Integrative Structural and Computational Biology, The Scripps Research Institute, La Jolla, CA, USA; 4 Micelio, Antwerp, Belgium

## Abstract

The accelerating growth of genomic and proteomic information for *Chlamydia* species, coupled with unique biological aspects of these pathogens, necessitates bioinformatic tools and features that are not provided by major public databases. To meet these growing needs, we developed ChlamBase, a model organism database for *Chlamydia* that is built upon the WikiGenomes application framework, and Wikidata, a community-curated database. ChlamBase was designed to serve as a central access point for genomic and proteomic information for the *Chlamydia* research community. ChlamBase integrates information from numerous external databases, as well as important data extracted from the literature that are otherwise not available in structured formats that are easy to use. In addition, a key feature of ChlamBase is that it empowers users in the field to contribute new annotations and data as the field advances with continued discoveries. ChlamBase is freely and publicly available at chlambase.org.

## Introduction

Chlamydiae are major human and veterinary pathogens; *Chlamydia trachomatis* is the most common bacterial sexually transmitted infection, responsible for millions of new infections every year (1–3). *C. trachomatis* was one of the early bacterial genomes to be fully sequenced (4), and a large number of *Chlamydia* species and clinical strains have since been sequenced. *Chlamydia* species possess a high extent of sequence homology and genomic synteny. This genomic information represents a rich resource for the research community, yet current tools for bioinformatic analysis of these data are inadequate and do not keep pace with the rapid growth of research in the field (5–7).

Model organism databases (MODs) are organized repositories of the knowledge accumulated over years of collective research efforts into the basic biology of an organism. A research community with a MOD has a central convergence point, with knowledge integrated into a user interface that dramatically reduces the friction involved with integration of genomic and proteomic data. MODs are accessed by individual scientists through web interfaces, as well as by bioinformatics analyses through application programming interfaces and downloadable data files. Examples of highly utilized MODs include FlyBase, Mouse Genome Database, *Saccharomyces* Genome Database, WormBase, EuPathDB and the Zebrafish Information Network (8–10).

For many research communities, including *Chlamydia*, highly developed MODs are not possible due to financial constraints that limit web development and biocuration efforts. The data generated by these smaller research communities fit into the `long-tail’ category of data that is extremely useful, but difficult to access and compute on, as it has not been integrated into structured data architectures. Furthermore, comprehensive genomic and proteomic data sets are frequently siloed within published tables or supplemental data and therefore inaccessible to bioinformatic databases. To unlock this knowledge from the literature corpus, a community-driven annotation model must be employed, wherein basic researchers are encouraged and empowered to contribute to the annotation effort through a powerful user interface.

The non-repetitive and concise genomes of *Chlamydia* offer a promising opportunity for capturing the complete genomic and proteomic information generated by this rapidly growing research community. To provide both a MOD and a powerful tool for the *Chlamydia* field to curate these data, we created ChlamBase (chlambase.org). ChlamBase was designed as a user-friendly platform that integrates data uniquely valued by the *Chlamydia* research community and allows for reference-based curation and annotation of knowledge not accessible in other public databases. The ChlamBase database was built using the WikiGenomes.org application codebase, which in turn is populated by data loaded from Wikidata (11). Wikidata (wikidata.org) is a community-curated database that is maintained by the Wikimedia Foundation and adheres to the same free and open principles of Wikipedia (12).

## Results

### Application architecture

To build ChlamBase, we leveraged the basic architecture of WikiGenomes.org (11), the genomic data web application for viewing and crowdsourcing bacterial genomic annotations in Wikidata (12–16). ChlamBase was built with AngularJS, a web framework for dynamic web applications, and Django, an open-source Python framework for running web servers. The National Center for Biotechnology Information (NCBI) is the authoritative source for all sequence data in ChlamBase, and we chose to initially load data for four widely studied *Chlamydia* reference genomes: *C. trachomatis `*434/BU' and *C. trachomatis `*D/UW-3/CX', *Chlamydia muridarum `*Nigg' and *Chlamydia pneumoniae CWL029*. Due to inconsistencies in the way NCBI stores and annotates plasmid data, we were only able to initially load plasmid data for *C. muridarum `*Nigg'. We will work toward adding more plasmids and whole genome sequences as the data become more available in NCBI, and the needs of the ChlamBase user community grow.

While the application is based on the prokaryotic focused WikiGenomes application, ChlamBase focuses on the specific needs and data types that are relevant to the chlamydial research community. To meet these needs, ChlamBase utilizes a hybrid backend that draws data from both Wikidata and a local database. Community contributions to ChlamBase are preferentially written to Wikidata, utilizing the local database only when the content is incompatible with Wikidata due to either data license or scope.

### Gene and chromosome annotation in Wikidata

**Figure 1:**
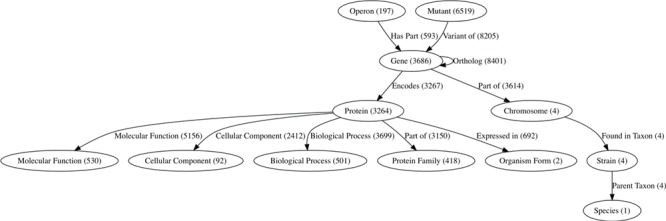
The semantic data model within Wikidata for ChlamBase content. Nodes in this graph represent classes of Wikidata items that are used in ChlamBase, with the count of each item class in parentheses. Edges in the graph represent statements in Wikidata that relate two items, with the total edge count between item types shown in parentheses.

ChlamBase manages and integrates chlamydial genomic and proteomic data in Wikidata using the WikidataIntegrator Python library (https://github.com/SuLab/WikidataIntegrator. We followed the same basic data model used in the WikiGenomes project (12), where each biological entity (e.g. gene, protein and regulatory element) is a Wikidata item, and the relationships/edges between those nodes are Wikidata properties (Figure 1).

### User interface

From the landing page, ChlamBase can be accessed through two points of entry: (i) selecting a *Chlamydia* genome by clicking on the visual phylogenetic tree or (ii) searching all available genomes for a gene or keyword of interest. If the user selects a genome, they will navigate to the organism browser page that displays a paginated list of all genes in that strain. Type-based entry into the search box provides the user with typeahead options for selecting a specific gene or a list of genes matching the search criteria. An `Advanced Search’ widget allows further faceting of genes based on gene annotations.

**Figure 2:**
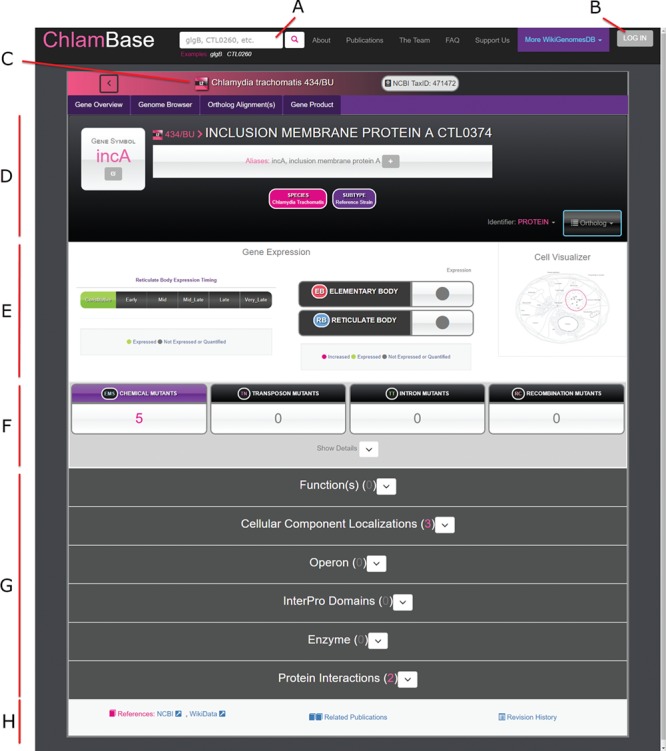
Overview of the ChlamBase gene page interface. (A) Search box to query the ChlamBase database for genes and keywords. (B) Log in button that redirects the user to a Wikimedia.org login for Wikidata account authorization and to allow the user to make edits or annotations using their credentials. (C) Display of the *Chlamydia* species and/or strain the current gene belongs to with strain-specific icons. (D) Key identifiers for the current gene, including gene name, gene symbol, locus tag and aliases. (E) Gene expression module showing the timing of expression (life cycle period or constitutive), expression in either chlamydial cell form (elementary body or reticulate body) and a cell localization diagram. (F) Mutant annotations produced by chemical, transposon, intron and recombination mutagenesis. (G) Annotation modules contain integrated and user-curated data for Gene Product, Ortholog(s), Ortholog Alignments, Expression Timing, Gene Ontology Annotations, Operon, InterPro Domains, Enzyme functions, Mutants, Protein Interactions and relevant Publications. All modules are loaded in expanded view upon page loading; module expansion and/or collapsing can be manually toggled by the user. (H) The gene page footer contains links to corresponding reference entries in NCBI and Wikidata, related publications identified by locus tag and species co-mention in the text and a link to the revision history in Wikidata.

Once a user selects a gene, they are navigated to the primary state for viewing content in ChlamBase—the gene page (Figure 2). ChlamBase gene pages were built to capture important gene and protein information and display them in a streamlined manner to the user. All gene pages contain important interface and structural features. In the page header, a search box allows for database-wide search queries for keywords or genes (Figure 2A). Below the header, the *Chlamydia* species/strain is displayed along with its NCBI taxonomy ID (Figure 2C). In this area, a toolbar allows the user to access an interactive genome browser, align orthologous sequences and view gene product (protein) information and tools. The Genome Browser tab in ChlamBase includes the open-source JavaScript genome browser JBrowse (17). JBrowse provides a fluid visual genome browsing experience with various user-selected feature tracks such as genes, operons and annotated mutations. Gene names are displayed along with the corresponding locus tags, gene aliases, and a downloadable sequence in FASTA format (Figure 2D). Below the gene name exists gene expression and protein localization data, structured in a graphical format (Figure 2E). The remainder of the gene page is structured around display-customizable annotation modules, including Gene Ontology Annotations, Operon, InterPro Domains, Enzyme Functions and Host–Pathogen Interactions (Figure 2G). Several of these modules are described in more detail in the sections below. The bottom of the gene page contains information for external reference links to NCBI and Wikidata, a list of publications related to the current gene, and a revision history list.

### Ortholog modules

Of particular importance to the *Chlamydia* research community is the orthologous nature of many genes in these genomes. Currently, bioinformatic tools for identifying and navigating between orthologous genes between *C. trachomatis* strains and *Chlamydia* species do not exist. Compounding the problem is that the locus tags for *C. trachomatis* D/UW-3 are widely used by the field, due to this strain being the first fully sequenced *Chlamydia* genome (4), whereas experimental genetic and proteomic data is more commonly generated in the *C. trachomatis* 434/BU strain. Furthermore, discovered functions of a gene product in one strain might also be relevant in the same, orthologous gene in another. Mass genome discovery requires a centralized annotation process that can be extended to the same gene in the other strains by a single form.

To address these issues, we first calculated sequence similarity for all pairs of genes across all four *Chlamydia* strains. We then assigned orthology based on a reciprocal best match followed by manual review. The complete procedure is described in https://github.com/SuLab/WikiGenomesBase/tree/master/scripts/blast/README.

**Figure 3:**
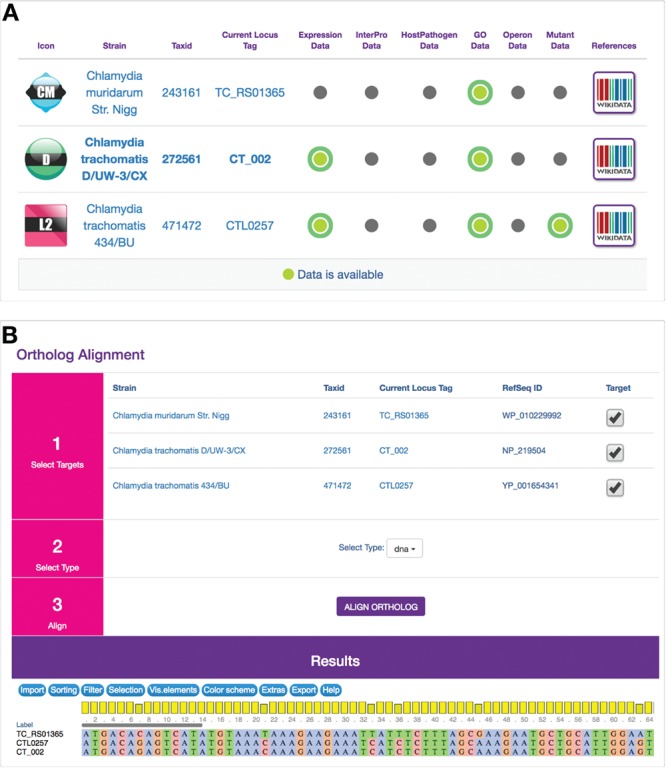
Ortholog and Ortholog Alignment modules. (A) Computationally derived orthologous genes from other *Chlamydia* genes in ChlamBase are displayed in a table along with the current gene. The table displays NCBI taxonomy IDs for orthologous genes, their locus tags (clicking on them redirects the user to those genes) and binary displays (by green or gray dots) of whether annotations are available in current/ortholog genes for Gene Expression, InterPro Domains, Host–Pathogen Interactions, Gene Ontology Annotations, Operons and Mutants. References open up Wikidata pages for that gene. (B) Ortholog Alignment tool uses the MSAViewer widget (19) to display DNA or amino acid alignments for orthologous genes or proteins, toggle species/strains to use for alignments and download aligned sequence data in a FASTA-compatible window.

ChlamBase utilizes *Chlamydia* orthology in two modules: Ortholog and Ortholog Alignment(s). The designed Ortholog window displays ChlamBase genes orthologous to the one loaded on the current gene page, arrayed in a table along with at-a-glance values for whether those genes contain annotations for Gene Expression Data, InterPro Domains, Host–Pathogen Interaction Data, Gene Ontology Annotations, Operons and Mutants (Figure 3A). The Ortholog Alignment component allows the user to view and download DNA or amino acid sequence alignments for orthologous genes and proteins (Figure 3B). This module delegates multiple alignments to the external web service MUSCLE (18) and subsequently displays the results in the JavaScript widget MSAViewer for interactive navigation of aligned sequences (19).

### Gene expression timing module

Gene transcription information is an example of data that is available in the literature, broadly useful to the *Chlamydia* research community, yet not available in existing public databases. As a first step toward unlocking these data from publication supplemental spreadsheets and making them available to users, we extracted the transcriptional microarray data for *C. trachomatis* L2/434/Bu (20) and converted the temporal assignments into a structured data table (Figure 2E). Since raw data are outside the scope of Wikidata, these values are stored locally to ChlamBase. All *C. trachomatis* L2 genes with assigned data in that publication are represented in the ChlamBase module using the same descriptors and a reference link to the parent publication in PubMed. Ongoing and future efforts will extract similar transcriptomic data for *C. trachomatis* D/UW-3 (21) and other studies as they become available.

**Figure 4:**
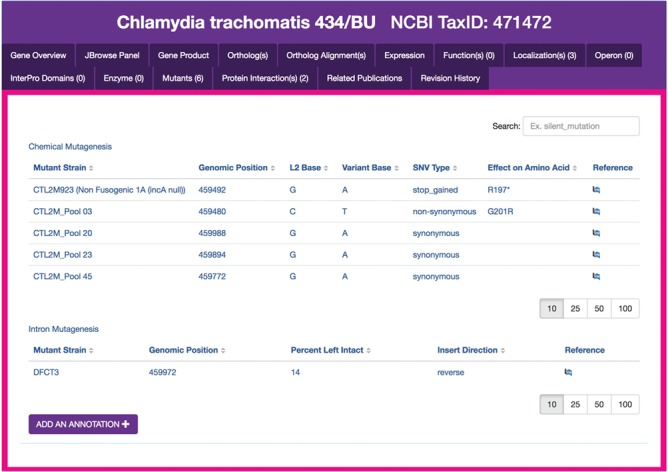
*Chlamydia* mutants module. Sample display of mutants for CTL0374 IncA, containing five mutant strains generated by chemical mutagenesis. The tabular view of chemical mutant strains displays strain names, genomic positions of mutations, the L2 reference and variant bases, SNV type, effects of each mutation on the respective amino acid sequences and a link to the publication reference. `Add an annotation’ opens a form for the submission of new mutant entries to ChlamBase.

### Mutants module

Another example of genomic data that is important to the research community but unavailable on public databases is the existence of *Chlamydia* mutant strains, including those generated from genome-wide mutagenesis screens, targeted gene insertions and transposon mutagenesis (22–24). We extracted the set of chemically derived mutations generated in *C. trachomatis* L2/434/Bu from a study that investigated the effects of gene knockouts across the genome (22). We created a structured version of the data contained within a supplemental data file (stored locally in ChlamBase) and display these data in the Mutants module of ChlamBase (Figure 4). On each gene page, all single-nucleotide variant (SNV) mutants described in this study are displayed in a tabular format, along with mutant strain names, the genomic position of the mutations, the reference and variant bases for each mutation, the SNV type, the effect of the mutation on the amino acid sequence and a link to the publication reference on PubMed or FigShare. Additionally, an embedded search box lets the user filter mutant strains for a particular type or keyword (e.g. non-synonymous or stop).

### Host–Pathogen Interactions module

**Figure 5:**
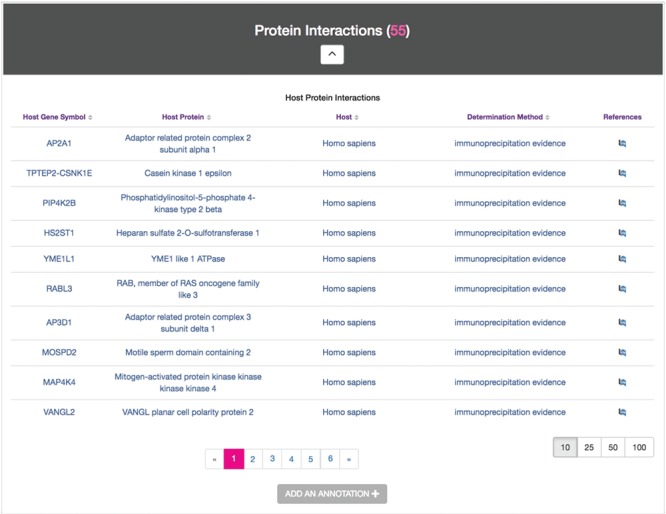
Host–Pathogen Interactions module. Sample information for CTL0260 IncV, containing a host protein found to interact with IncV, its corresponding gene symbols, host species, the determination methods used for this evidence and links to the publication references.

The Host–Pathogen Interactions module displays experimentally determined targets of secreted *Chlamydia* proteins. In an effort to capture the accelerating volume of *Chlamydia*–host interaction data (25), ChlamBase extracts proteomic data that is abundant in the scientific literature but not yet accessible in major public databases. The Host–Pathogen Interactions module displays annotations for host protein(s) demonstrated to interact with the protein encoded by the gene described on each gene page (Figure 5).

### Community contribution

**Figure 6:**
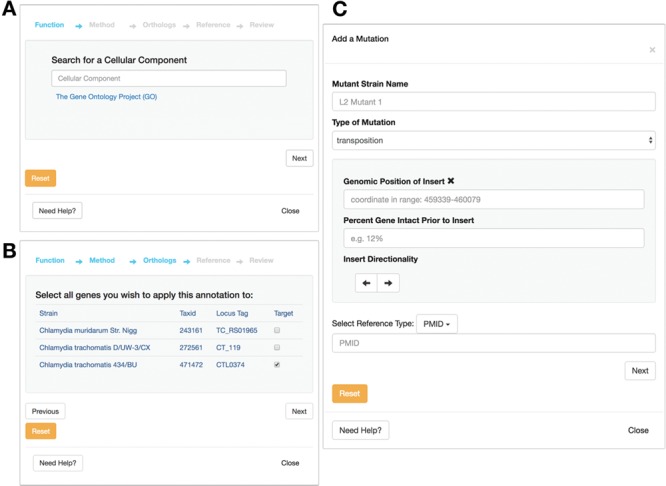
Annotation submission forms for ChlamBase. The GO form prompts the user to submit information for (A) GO term, typeahead enabled and the method used for determination (i.e. inferred from experiment and inferred from sequence orthology); (B) the ability to map the GO annotation to orthologous strains, and the PubMed reference used as the experimental basis for this annotation. (C) Mutant annotation form asks the user to submit information for: (i) mutant strain name, (ii) mutation type (i.e. chemical mutagenesis or transposon mutagenesis) and (iii) the PubMed or FigShare reference that serves as the evidence for the annotation. Additional mutant information is available for entry depending on the mutation type chosen by the user.

In addition to allowing users to consume data on *Chlamydia*, a central feature of ChlamBase is to also allow users to contribute new knowledge back to this community resource. Any member of the research community can deposit new genomic or proteomic information, which will then be immediately visible for other users to see. To minimize friction for data entry, ChlamBase offers user-friendly annotation wizards that guide users through the annotation process in a stepwise format. Importantly, these annotation wizards require the user to include a reference (in the form of a PubMed ID or a FigShare DOI) that describes the source of the annotation, and this provenance is stored as a core part of the annotation. The ability for users to contribute new information applies to data in most ChlamBase modules, including gene names and aliases, Gene Ontology (GO) terms (Figure 6A and B), Operon, Mutants (Figure 6C) and Host–Pathogen Interactions.

## Discussion

ChlamBase was built with the goal of enabling and empowering the *Chlamydia* research community to collaboratively organize genomic and proteomic information. Since the first sequenced *Chlamydia* genome in 1998 (4), the field has grappled with effective tools to digest and analyze large data. As the field embarks on an accelerating omic age (5, 7), the rapid accumulation of big data sets will require an intelligent online repository to aggregate and promote interactions with this information. Existing broad-spectrum databases that serve basic information management needs for every research community are important resources, but they fail to deliver focused, community-specific solutions that are tailored to organism-specific use cases. An even greater shortcoming of major databases is that they do not capture the `long tail’ of genomic and proteomic information that is frequently buried within supplemental data of important publications (12). Thus, perhaps the greatest potential of ChlamBase is in empowering researchers to capture data from publications or supplemental data, which big data warehouses have yet to acquire, and present it in a manner that is viewable and interactive.

While the design of the ChlamBase user interface revolved around filling a need for the *Chlamydia* research community, the overall application architecture reflects a broader interest in open data. We have had a long-standing interest in using Wikidata to integrate biomedical data, primarily through importing large structured databases and data sets (11–13, 16). While Wikidata is in principle also open to community edits by individuals, in practice it is highly unlikely that biomedical domain experts would contribute through the standard Wikidata interfaces. We view ChlamBase as a thin web interface that serves as a bidirectional translation layer between Wikidata and expert *Chlamydia* researchers. We believe that ChlamBase (and other similar applications) will play key roles in enabling domain experts to contribute structured knowledge and in storing that knowledge in a fully open community resource.

## Notes

**Database URL:** https://chlambase.org/
