# ChlamBase: a curated model organism database for the *Chlamydia* research community

**DOI:** 10.1093/database/baz091

**Published:** 2019-06-18

**Authors:** Tim Putman, Kevin Hybiske, Derek Jow, Cyrus Afrasiabi, Sebastien Lelong, Marco Alvarado Cano, Gregory S Stupp, Andra Waagmeester, Benjamin M Good, Chunlei Wu, Andrew I Su

**Affiliations:** 1 Ontology Development Group, Library, Oregon Health and Science University, Portland, OR, USA; 2 Division of Allergy and Infectious Diseases, Department of Medicine, University of Washington, Seattle, WA, USA; 3 Department of Integrative Structural and Computational Biology, The Scripps Research Institute, La Jolla, CA, USA; 4 Micelio, Antwerp, Belgium

This manuscript has been amended to include additional authors who were inadvertently omitted.

